# Foxo1‐induced miR‐92b down‐regulation promotes blood‐brain barrier damage after ischaemic stroke by targeting NOX4

**DOI:** 10.1111/jcmm.16537

**Published:** 2021-05-06

**Authors:** Jian Shen, Ganglei Li, Yu Zhu, Qingsheng Xu, Hengjun Zhou, Kangli Xu, Kaiyuan Huang, Renya Zhan, Jianwei Pan

**Affiliations:** ^1^ Department of Neurosurgery The First Affiliated Hospital College of Medicine Zhejiang University Hangzhou China

**Keywords:** blood‐brain barrier, Foxo1, ischaemic stroke, miR‐92b, NOX4

## Abstract

The blood‐brain barrier (BBB) damage is a momentous pathological process of ischaemic stroke. NADPH oxidases 4 (NOX4) boosts BBB damage after ischaemic stroke and its expression can be influenced by microRNAs. This study aimed to probe into whether miR‐92b influenced the BBB damage after ischaemic stroke by regulating NOX4 expression. Here, miR‐92b expression was lessened in the ischaemic brains of rats and oxygen‐glucose deprivation (OGD)‐induced brain microvascular endothelial cells (BMECs). In middle cerebral artery occlusion (MCAo) rats, miR‐92b overexpression relieved the ameliorated neurological function and protected the BBB integrity. *In vitro* model, miR‐92b overexpression raised the viability and lessened the permeability of OGD‐induced BMECs. miR‐92b targeted NOX4 and regulated the viability and permeability of OGD‐induced BMECs by negatively modulating NOX4 expression. The transcription factor Foxo1 bound to the miR‐92b promoter and restrained its expression. Foxo1 expression was induced by OGD‐induction and its knockdown abolished the effects of OGD on miR‐92b and NOX4 expressions, cell viability and permeability of BMECs. In general, our findings expounded that Foxo1‐induced lessening miR‐92b boosted BBB damage after ischaemic stroke by raising NOX4 expression.

## 1 INTRODUCTION

Stroke is an acute cerebrovascular disorder, which is caused by abnormal blood supply, such as ischaemia or haemorrhage and resulted in long‐term neurological injury.^1^, ^2^ Approximately, 795 thousand people suffer from a new or recurrent stroke annually and the most are ischaemic strokes.^3^ Nevertheless, the pathogenesis of ischaemic stroke is still largely unknown. Recent studies have expounded that the blood‐brain barrier (BBB) damage is a momentous pathological process of ischaemic stroke.^4^ Physiologically, the BBB is a unique microvascular structure composed of brain microvascular endothelial cells (BMECs), astrocytes and pericytes in the neurovascular unit (NVU), which precisely controls cerebral homeostasis and maintain neurological function.^5^ After ischaemic stroke, the integrity of BBB is impaired due to raised permeability of BMECs, and a large number of harmful substances and fluids enter the brain parenchyma, eventually resulting in brain oedema and neurological deficit.^6^, ^7^ Hence, probing into the mechanism of BBB damage is momentous for the understanding of ischaemic stroke pathogenesis.

NADPH oxidases (NOXs) are the major enzymes for producing reactive oxygen species (ROS).^8^ There are four subtypes of NOXs (NOX1, NOX2, NOX3 and NOX4) that have been identified in rodents. Among these four NOXs subtypes, NOX4 is the predominant subtype expressed in BMECs and its expression can be induced after ischaemic stroke.^9^, ^10^ Further studies expounded that NOX4 is bound up with the BBB damage in ischaemic stroke. In the rat model of ischaemic stroke, the NOX4 inhibitor treatment can relieve the BBB damage.^11^ In the mouse model of ischaemic stroke, the deficiency of NOX4 in BMECs can protect the integrity of BBB.^10^, ^12^ However, the upstream mechanism of NOX4 expression in regulating BBB integrity in ischaemic stroke has not been fully elucidated.

**FIGURE 1:**
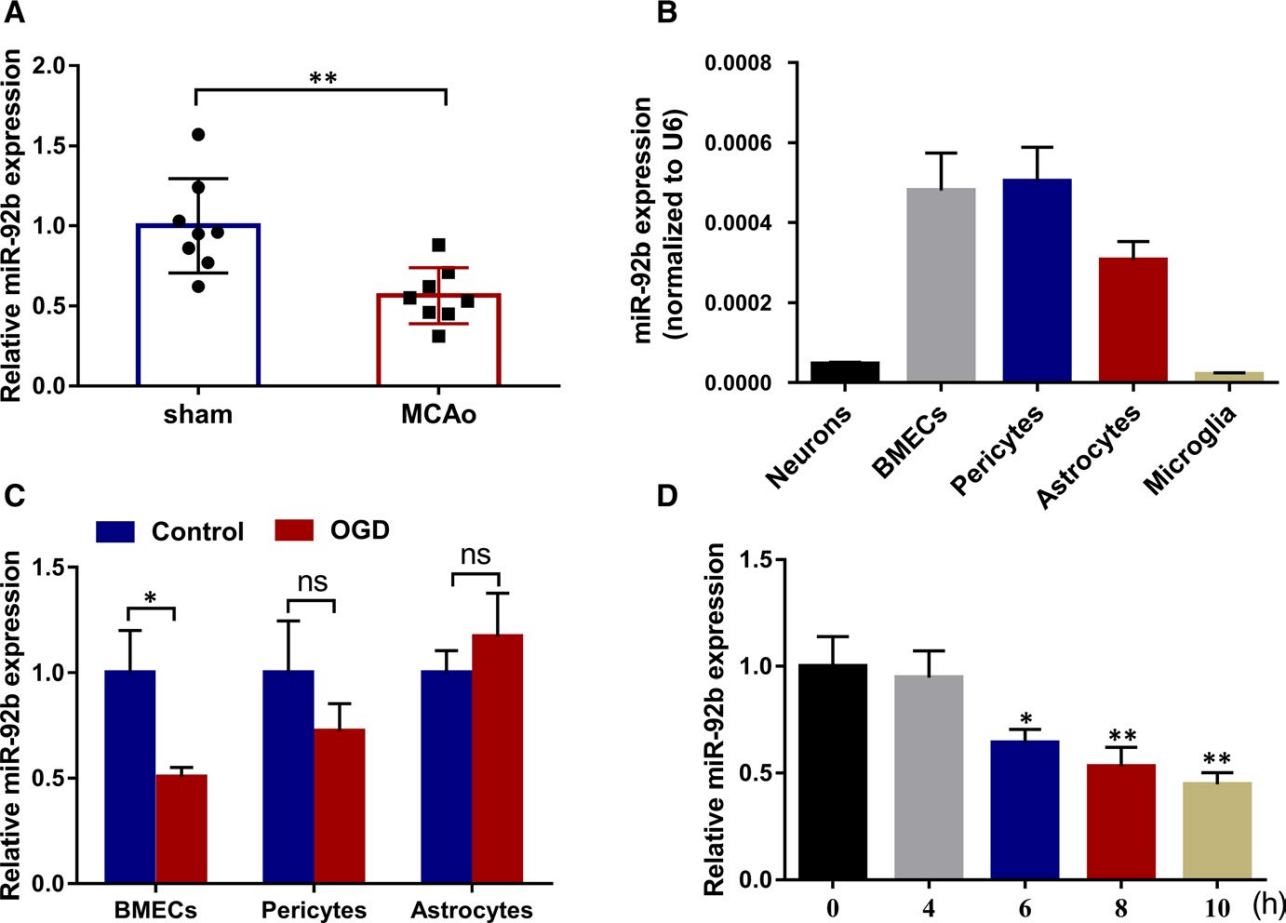
The expression of miR‐92b in the ischaemic brain tissues and OGD‐induced BMECs. A, The expression of miR‐92b in the brain tissues of sham and MCAo rats was tested by qRT‐PCR. 8 rats per group. **P < 0.01 vs sham. SD (Sham): 0.29; SD (MCAo): 0.17. B, The expressions of miR‐92b in the rat primary BMECs, neurons, pericytes, astrocytes and microglia were tested by qRT‐PCR. SD (neurons): 3.85E‐06; SD (BMECs): 9.36E‐05; SD (pericytes): 8.50E‐05; SD (astrocytes): 4.57E‐05; SD (microglia): 3.97E‐06. C, BMECs, pericytes and astrocytes were induced by OGD for 6 h, respectively. The expression of miR‐92b in cells was tested by qRT‐PCR. *P < 0.05 vs Control; ns: no significant difference. SD (BMECs‐control): 0.20; SD (BMECs‐OGD): 0.05; SD (pericytes‐control): 0.26; SD (pericytes‐OGD): 0.13; SD (astrocytes‐control): 0.11; SD (astrocytes‐OGD): 0.21. D, Immortalized human BMECs (hCMEC/D3) were induced by OGD for 0, 4, 6, 8 or 10 h. The expression of miR‐92b in cells was tested by qRT‐PCR. *P < 0.05 or **P < 0.01 vs 0 h. SD (0 h): 0.14; SD (4 h): 0.13; SD (6 h): 0.06; SD (8 h): 0.09; SD (10 h): 0.06. Data are represented as the mean ± SD of three independent assays

MicroRNAs (miRNAs) are a class of short non‐coding RNAs and can regulate multiple cell processes, such as cell apoptosis and cell autophagy.^13^, ^14^, ^15^ miRNAs usually exert biological functions by binding to the seed region on the 3′‐untranslated region (3′‐UTR) of target mRNA.^16^ It has been proven that miRNAs can target NOX4 and modulate its expression in multiple diseases.^17^, ^18^ In ischaemic stroke, Zhong Liu *et al* reported that the abnormal expression of NOX4 after cerebral ischaemia may be regulated by its upstream miRNA in view of the bioinformatics and miRNA microarray, but this has not been further confirmed.^19^ In our preliminary work, we predicted the potential miRNAs that bound to the 3′‐UTR of NOX4 using the bioinformatics software (microRNA.org‐Targets and Expression: http://www.microrna.org/microrna/getGeneForm.do). The results expounded that both the human, rat and mouse NOX4 3′‐UTR had the potential binding sites of miR‐92b and miR‐363. We then probed into the expression profiles of these two miRNAs in ischaemic stroke. We discovered that miR‐92b expression was lessened in the ischaemic brains of rats (Figure 1A), whereas miR‐363 was not significantly changed in the brains after ischaemia (data were not shown). Nevertheless, whether miR‐92b can target NOX4 and affect the BBB damage in ischaemic stroke remains unknown.

This study was designed to probe into whether miR‐92b influenced the BBB damage in ischaemic stroke by regulating NOX4. Our *in vivo* experiments in a rat model of ischaemic stroke expounded that the overexpression of miR‐92b protected the integrity of BBB in ischaemic stroke. The *in vitro* studies expounded that the lessening of miR‐92b was induced by Foxo1 and that miR‐92b regulated the viability and permeability of oxygen‐glucose deprivation (OGD)‐induced BMECs by negatively modulating NOX4 expression.

## 2 MATERIALS AND METHODS

### 2.1 The rat model of ischaemic stroke

Male Sprague Dawley rats (8‐9 weeks old, 300 ± 20 g) were purchased from Shanghai Experimental Animal Center, Chinese Academy of Science. The rats were housed in the ventilated cages and supplied with a standard diet. All animal experimental producers were approved by the Animal Ethics Committee of The First Affiliated Hospital, College of Medicine, Zhejiang University.

The rat model was structured using the middle cerebral artery occlusion (MCAo) method in view of the previously described methods.^20^ Briefly, the rats were anaesthetized intraperitoneally using 40 mg/kg pentobarbital. Then, the right common carotid artery (CCA), external carotid artery (ECA) and internal carotid artery (ICA) were separated through a midline incision. The ECA was ligated with a 6‐0 nylon suture. The CCA was inserted a 4‐0 poly‐L‐lysine‐coated nylon suture (Sunbio Biotech Co Ltd, China) to ICA and then transferred to 18 mm from the origin of the middle cerebral artery.^21^ Body temperature was retained at 37 ± 0.5°C during the procedures. The rats in the sham group were received identical procedures but without the filament insertion.

### 2.2 Injection of adeno‐associated virus (AAV) vector

The AAV vector expressing miR‐92b was synthesized by Genomeditech (China). Then, 2.5 µL AAV vector (8.1 × 10^12^ µg/mL) was injected intracerebrally into the subdural brain of rats. One week after AVV vector injection, rats were subjected to structure ischaemic stroke models as mentioned above.

### 2.3 Assessment of neurological score

Rats were monitored by a video camera after MCAo surgery. 22 hours after MCAo, the neurological deficit of rats was assessed by a 5‐point scale method. The neurological scores were tested by 5 grades (0‐4). The 5‐point scale method is as follows: 0: represents normal motor function; 1: represents flexion of contralateral torso and forelimb upon lifting the whole animal by the tail; 2: represents circling to the contralateral side but normal posture at rest; 3: represents leaning to the contralateral side at rest; 4: represents no spontaneous motor activity, and the higher‐grade indicates a more serious neurological deficit.^22^

### 2.4 Measurement of brain infarct volume

Brain infarct volume was tested in view of 2, 3, 5‐triphenyl tetrazolium chloride monohydrate ^23^ assay as previously described.^24^ The brain tissues were cut into 2 mm‐thick sections (these sections were from equivalent areas of the brain) and then immersed into a TTC solution (2%). Later, the sections were fixed with 10% formaldehyde for nearly 24 hours. The infarcted brain tissues appear pale, whereas the non‐infarcted areas appear red.^25^ Evaluation of infarct volume was tested by an electron microscope and analysed by Image J software (NIH, Bethesda, MD, USA), and the cerebral infarct volume of the rats was tested by the analysis of Image J software (NIH, Bethesda, MD, USA). The cerebral infarct volume is regarded as the percentage of the infarct area/the area of the ipsilateral hemisphere at the coronal section of the optic chiasma.

### 2.5 Measurement of brain water content

Rats were sacrificed and the brains were gathered. Bilateral cerebral hemispheres were segregated and weighed. After being dried at 100°C for nearly 48 hours, the bilateral hemispheres of the brains were weighed again. The brain water content (%) = (wet weight‐dry weight)/wet weight × 100%.^25^

### 2.6 Evans blue assay

The Evans blue staining, a marker of albumin leakage, was employed to test the permeability of BBB. Evans blue (EB, 2% in PBS, 4 mL/kg) was injected into rats through the tail vein.^26^ After 3 hours, the rats were transcardially perfused with cold saline to remove intravascular EB and the blood concentration of EB was not measured. After being sacrificed by cervical dislocation, the bilateral cerebral hemispheres were weighed. One side hemisphere was applied to make 10‐20 µm‐thick frozen sections. The sections were observed under a microscope. The other side hemisphere was weighed, sheared and then incubated in the dimethylformamide solution (1 mL/100 mg) at 60°C for nearly 24 hours. After centrifugation, the content of Evans blue in the supernatant was tested by a microplate reader (Biorad, USA) at 623 nm.^25^

### 2.7 Detection of reactive oxygen species

The production of reactive oxygen species (ROS) was tested by the DCFH‐DA method.^27^ The brain tissues were digested to single‐cell suspensions. Then, cells were resuspended with DCFH‐DA solution (1 × 10^7^ cells/mL). After 0.5 hour incubation, cells were gathered and centrifuged. Cells were washed with PBS buffer, and then the ROS production was tested by a microplate reader (Biorad, USA). The excitation and emission wavelengths for detection were 500 and 525 nm, respectively.

### 2.8 Detection of superoxide dismutase activity

The activity of superoxide dismutase (SOD) was tested by the Total Superoxide Dismutase Assay Kit with WST‐8 (Beyotime, China). Briefly, 100 µL sample preparation solution was applied to 10 mg brain tissues, and then the tissues were homogenized at 4°C, followed by nearly 5 minutes centrifugation. Then, 160 µL WST‐8 solution and 20 µL reaction starting working solution were applied to 20 µL supernatant. After nearly 30 minutes incubation at 37°C, the absorbance at 450 nm was tested by a microplate reader (Biorad, USA).

### 2.9 Cell culture and OGD‐induction

The primary BMECs, pericytes, microglia, neurons and astrocytes were segregated from the brain tissues of neonatal rats.

The primary BMECs were isolated from the brain tissues of neonatal rats. The specific procedure was as follows: After removing the cerebellum, intercerebral, brain stem and pia mater, we rinsed the cerebral cortex with PBS and then divided it into small pieces and digested it with 0.1% collagenase II (Gibco). The digested mixture was filtered with a strainer (pore size was 178 μm) and centrifuged at low speed for 7‐9 minutes at 4°C. The obtained precipitate was resuspended in BSA and continued to centrifuge at 4°C for 15‐25 minutes. Then, we digested the lower layer of capillaries with 0.1% collagenase II/dispase (Solarbio) at room temperature for nearly 1 hour and left it for about 5 minutes after centrifugation and then applied DMEM (Gibco) to dissolve the precipitate gathered above and continued to centrifuge for 15‐25 minutes and repeated the operation 2‐3 times to gather the final primary BMECs. Cells were cultured in DMEM medium with the addition of foetal bovine serum (FBS, 10%) and incubated at 37°C and 5% CO_2_.

The primary pericytes were segregated from the brain tissues of neonatal rats. The specific procedure was as follows: The bones of rat embryos were incubated in trypsin solution for nearly 15 minutes at room temperature. During the entire incubation, we shook the above solution vigorously every 5 minutes. Then, the solution was filtered through a cell strainer (40 μm) and transferred to another test tube.

The primary microglia were segregated from the brain tissues of neonatal rats. The specific procedure was as follows: First, we prepared a mixed glial culture from the cerebral cortex of a rat and put it in DMEM with the addition of 10% FBS for 12‐23 days. Then, we gathered the floating microglia on the mixed glial cell culture and then cultured them in DMEM medium complemented with 10% FBS and incubated at 37°C and 5% CO_2_, and the isolation of primary neurons was conducted in view of the previously described methods.

The primary astrocytes were isolated from the brain tissues of neonatal rats. The specific procedure was as follows: The brain tissues of the rats gathered after centrifugation were resuscitated in ice‐cold DMEM. Then, we applied to pipet to mince the tissues to segregate the cells. After filtering the cells with a filter (40 microns), we put them in a DMEM medium with the addition of 10% FBS and incubated at 37°C and 5% CO_2_.

Then, the BMECs, pericytes and astrocytes were exposed to transient OGD for 6 hours. In brief, cells were cultured in the glucose‐free DMEM medium and incubated at 37°C, 95% N_2_ in an anaerobic incubator (GeneScience Pharmaceuticals Co., Ltd) and 5% CO_2_. 6 hours later, cells were cultured in the glucose‐containing DMEM medium and incubated at 37°C, 95% air and 5% CO_2_. Control cells were cultured in the glucose‐containing DMEM medium and incubated at 37°C, 95% air and 5% CO_2_.

Immortalized human BMECs (hCMEC/D3) was purchased from Merk Millipore (Germany). The hCMEC/D3 cells were cultured in EndoGRO‐MV complete culture media (Merck spa, Italy) containing 1 ng/mL fibroblast growth factor and 1% penicillin‐streptomycin. Then, hCMEC/D3 cells were exposed to transient OGD for 0, 1, 2, 3, 4, 5 and 6 hours.

### 2.10 Cell transfection

The overexpression vectors (miR‐92b mimic, pcDNA‐NOX4 and pcDNA‐Foxo1) and silence vectors (miR‐92b inhibitor, si‐NOX4 and si‐Foxo1) were synthesized by the GenePharma (China). The negative control of miR‐92b mimic and miR‐92b inhibitor was mimic‐NC and inhibitor‐NC, respectively. The negative control of pcDNA‐NOX4 and pcDNA‐Foxo1 was pcDNA‐NC. The negative control of si‐NOX4 and si‐Foxo1 was si‐NC. These vectors were transfected into hCMEC/D3 cells using Lipofectamine2000 (Invitrogen, USA). The corresponding gene sequences are presented in Table 1.

**TABLE 1:** All the sequences used in this study

Name	Sequence(5′‐3′)
miR‐92b	GTCGTATCCAGTGCAGGGTCCGAGGTATTCGCACTGGATACGACGGAGGCCGG
miR‐92b Forward primer	TATATTGCACTCGTCCCGGC
miR‐92b Reverse Primer	CTAGTGCAGGGTCCGAGGTATT
miR‐92b mimics NC Forward	CUCCUGAUCACUUGTGUTACG
miR‐92b inhibitor NC	CAGUACUUAUGUGUAGUACAA
miR‐92b inhibitor	GGAGGCCGGGACGAGUGCAAUA
U6 Forward	TCGCTTCGGCAGCACATA
U6 Reverse	TTCACGAATTTGCGTGTC
Foxo1 Forward primer	5′‐TTAGCCAGTCCAACTCGG‐3′
Foxo1 Reverse primer	5′‐TCTTGACCATCCACTCGTA‐3′
Si‐NOX4	AACGAAGGGGUUAAACACCUC
Si‐NC	GGUTGATGGTGTCGGAGTAAG
NOX4 Forward primer	TGCCTGCTCATTTGGCTGT
NOX4 Reverse primer	CCGGCACATAGGTAAAAGGATG
GAPDH Forward primer	GAAGATGGTGATGGGATTTC
GAPDH Reverse primer	GAAGGTGAAGGTCGGAGT

### 2.11 Cell viability

The viability of hCMEC/D3 cells was tested by MTT kits (Beyotime, China). Briefly, hCMEC/D3 cells were seeded in a 96‐well plate (5 × 10^3^cells/well). Each well was added with 10 µL MTT solution and incubated in the EndoGRO‐MV complete culture media (Merck spa, Italy) for nearly 4 hours at 37°C, 5% CO_2_. Cell viability was tested at an absorbance of 570 nm by a microplate reader (Biorad, USA). Moreover, the EZViableTM calcein‐AM cell viability assay kit (Fluorometric) was also applied to test cell viability in view of the standard procedure of the reagent manufacturer.

### 2.12 Measurement of transendothelial electrical resistance and paracellular permeability

The *in vitro* model of BBB was established using monolayers of hCMEC/D3 cells as previously described.^28^ Cells were cultured in the DMEM medium with the addition of 10% FBS. Each transwell insert was precoated with poly‐l‐lysine and seeded with 6 × 10^4^ cells. Cells were cultured for 8 days at 37°C and 5% CO_2_. To assess alterations in ion flux through the endothelial monolayer, the transendothelial electrical resistance (TEER) values across endothelial monolayer were tested with an Endohm‐12 electrode chamber connected to an EVOM2 Epithelial Voltohmmeter (World Precision Instruments, USA). The final result was calculated as TEER in each group—TEER in blank control. Each experiment was repeated three times.

The 4.4 kD tetramethylrhodamine isothiocyanate (TRITC)‐dextran and 70 kD fluorescein isothiocyanate (FITC)‐dextran were utilized to test the paracellular permeability of hCMEC/D3 cells, as previously described.^25^ Briefly, hCMEC/D3 cells (2.5 × 10^5^) were seeded onto per chamber. The culture medium with the addition of TRITC‐dextran or FITC‐dextran was added into the luminal chamber. After 0, 1, 2 and 3 hours incubation, the fluorescence intensity of 50 µL culture medium from the abluminal chamber was tested by a microplate reader (Biorad, USA) to form a standard curve. The excitation wavelength was 485 nm, and the emission wavelength was 525 nm. The permeability of cells was calculated by testing the diffusion coefficient of TRITC or FITC from the luminal to the abluminal chamber.^28^

### 2.13 Haematoxylin‐eosin staining

Haematoxylin‐eosin (HE) staining was applied to appraise the pathological changes of brain tissues in rats. Briefly, the brain tissues were fixed with 4% formaldehyde and embedded in paraffin. Tissue sections with a thickness of 5 μm were then prepared for the HE staining. The observations were made under the microscope.

### 2.14 Dual‐luciferase reporter assay

The wild‐type (WT) sequence of NOX4‐3′‐UTR (NOX4‐3′‐UTR‐WT) and mutated (MU) sequence of NOX4‐3′‐UTR (NOX4‐3′‐UTR‐MU) were subcloned into the upstream of the luciferase reporter gene in a pGL3 plasmid (Promega, USA). Each recombinant vector was co‐transfected with miR‐92b mimic (or negative control: mimic‐NC) into 293T cells. Then, the luciferase activity was tested by the Dual‐Luciferase Reporter Assay System (Promega, USA).

The WT promoter sequence of miR‐92b (miR‐92b‐WT) and MU promoter sequence of miR‐92b (miR‐92b‐MU) were subcloned into the upstream of a luciferase reporter gene in a pGL3 plasmid. Each recombinant vector was co‐transfected with pcDNA‐Foxo1 (or negative control: pcDNA) into 293T cells. Then, the luciferase activity was tested by the Dual‐Luciferase Reporter Assay System (Promega, USA).

### 2.15 RNA extraction and qRT‐PCR

Total RNA was segregated from brain tissues or cells by Trizol Reagent (Invitrogen, USA). After the RNA was quantified by an agarose gel electrophoresis, 1000 ng RNA was applied to synthesize cDNA by cDNA Synthesis Kit (Takara, Japan). qRT‐PCR was conducted with SYBR^@^ Premix Ex TaqTM Kit (Takara, Japan) following its manufacturer's instruction. The relative expressions of genes were tested by the 2^−△△CT^ method. All experiments were replicated in triplicate. All primer sequences are shown in Table 1.

### 2.16 Western blot

For the protein extraction from tissues, RIPA lysis buffer (50 mmol/L Tris, pH 7.4, 150 mmol/L NaCl, 1% NP‐40 and 0.5% sodium deoxycholate) was added to the mortar and the tissues were thoroughly ground on ice, and then the supernatant obtained after centrifugation in a low‐temperature high‐speed centrifuge (Beckman, LE‐80K) at 4°C and 4500 g for 15 minutes was the protein sample. For the protein extraction from hCMEC/D3 cells, RIPA lysis buffer (50 mmol/L Tris, pH 7.4, 150 mmol/L NaCl, 1% NP‐40 and 0.5% sodium deoxycholate) was added to extract protein. Total protein concentrations were tested by the BCA Protein assay kit (Thermo Fisher Scientific, USA). Protein samples (25 μg) were segregated by 10% SDS‐PAGE gels, and an equal amount of proteins was transferred onto polyvinylidene difluoride (PVDF) membranes. Membranes were incubated with the polyclonal rabbit primary antibodies: anti‐NOX4 (Abcam, ab133303, 1:1000), anti‐ZO‐1 (Abcam, ab96587, 1:500), anti‐β‐actin (Abcam, ab8227, 1:1000), anti‐occludin (Abcam, ab216327, 1:250), anti‐VE‐cadherin (Abcam, ab205336, 1:1000) and anti‐Foxo1 (Abcam, ab179450, 1:500) overnight at 4°C and then incubated with corresponding horseradish peroxidase‐conjugated secondary antibody (Sigma Aldrich, AC111P, 1:10 000) for nearly 1 hour. Protein bands were visualized by a chemiluminescence kit (Thermo Scientific, USA). The experiments were repeated three times.

### 2.17 Chromatin immunoprecipitation (ChIP)assay

The ChIP assay was conducted using the Magna ChIP™G Tissue Kit (Magna, USA). hCMEC/D3 cells were fixed with 1% formaldehyde (Aladdin) for nearly 10 minutes at room temperature, and glycine was applied to stop cross‐linking. Then, cells were lysed with ChIP lysis buffer (Magna, USA). Cell lysates were sonicated to generate DNA fragments of 200‐1000 bp. The 20 µL lysate was transferred into a new EP tube as input. The remaining lysate was divided into three parts and incubated with the anti‐Foxo1, anti‐H3 or IgG at 4°C for nearly 24 hours to prepare the immunoprecipitated protein‐DNA complexes. After then, Protein A/G magnetic beads were applied to lysates and the lysates were washed with 100 μL ChIP wash buffer. The DNA was extracted from immunoprecipitated protein‐DNA complexes by the phenol‐chloroform method. The miR‐92b in DNA samples was tested by agarose gel electrophoresis. IgG and H3 were utilized as a negative and positive control of Foxo1, respectively.

### 2.18 Statistical analysis

All data were analyzed by Graphpad Prism 7.0 and expressed as mean ± standard deviation. Group differences were compared by Student's *t* test or one‐way ANOVA in which were applicable. A *P*‐value < 0.05 was considered statistically significant.

## 3 RESULTS

### 3.1 miR‐92b expression was lessened in the ischaemic brains of rats and OGD‐induced BMECs

To study the expression of miR‐92b in ischaemic stroke, we structured a rat model of ischaemic stroke by the MACo method and then tested the expression of miR‐92b in the ischaemic brains of rats. The results expounded that miR‐92b expression was lessened in the brains of MCAo rats compared with sham rats (Figure 1A). To probe into the potential role of miR‐92b in the brain, we tested the expression of miR‐92b in five types of cells from the rat brain. As displayed in Figure 1B, miR‐92b expressed in all of the five types of cells and predominantly expressed in BMECs, pericytes and the astrocytes, implying that miR‐92b might play a momentous role in protecting BBB integrity. Next, the effects of OGD‐induction on miR‐92b expression in BMECs, pericytes and astrocytes were tested. The results expounded that OGD‐induction lessened miR‐92b expression in BMECs, whereas had no significant effect on miR‐92b expression in pericytes and astrocytes (Figure 1C). Subsequently, we verified the effect of OGD‐induction on human BMECs (hCMEC/D3 cells). The results expounded that OGD‐induction lessened miR‐92b expression in hCMEC/D3 cells (Figure 1D). Besides, we tested the viability of the cells after 6‐10 hours of OGD treatment, and the results expounded that the cell viability was lessened gradually after 6 hours of OGD treatment, and the most obvious lessening in cell viability was 10 hours (Figure S1A and B). These results expounded that miR‐92b expression was lessened in the ischaemic brains of rats and OGD‐induced BMECs.

### 3.2 Overexpression of miR‐92b protected the BBB integrity of ischaemic stroke rats

**FIGURE 2:**
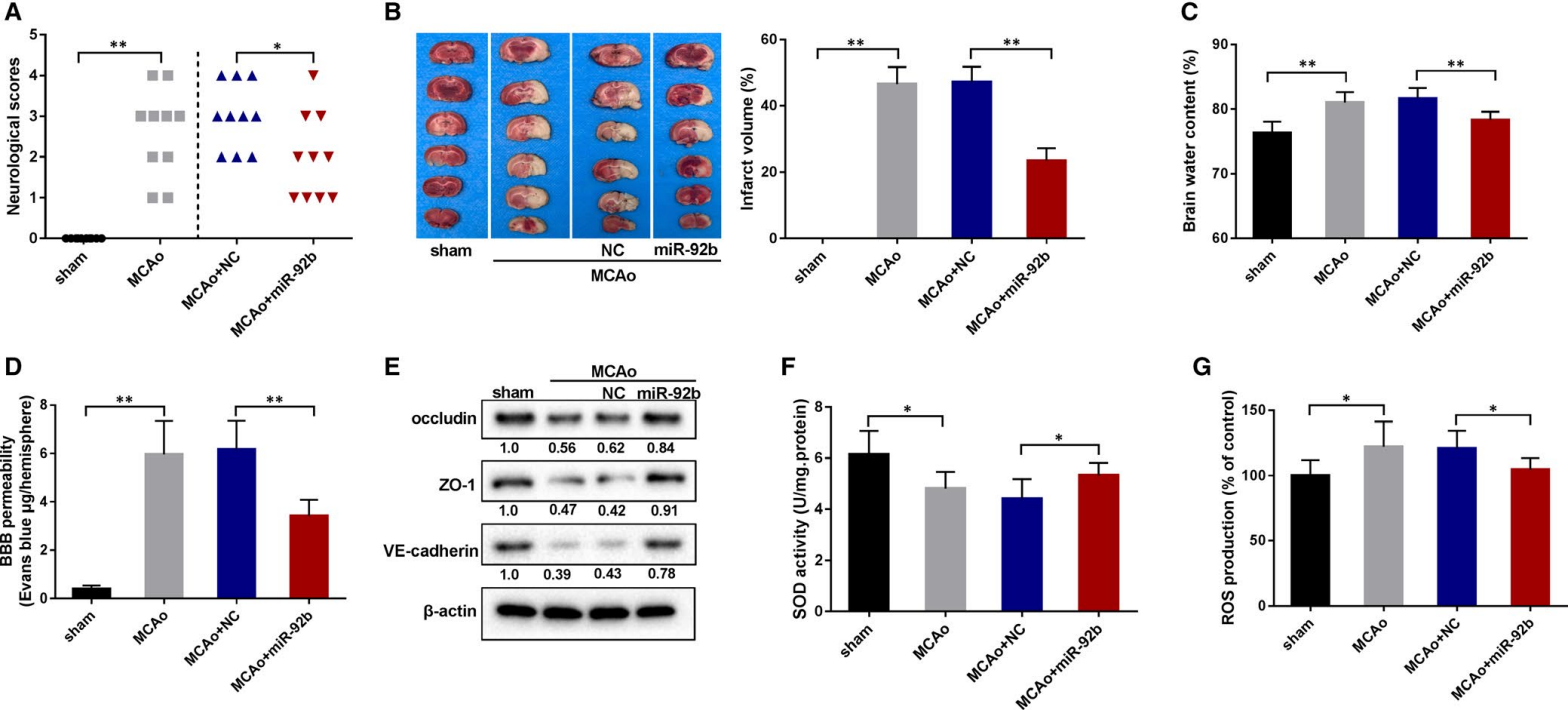
The effect of miR‐92b overexpression on the BBB in the rat model of ischaemic stroke. The MCAo rats were injected with an adeno‐associated virus expressing miR‐92b or negative control (NC). A, The behavioural score was assessed by a 6‐point scale method. 10 rats per group. SD (MCAo): 1.14; SD (MCAo + NC): 0.71; SD (MCAo + miR‐92b): 0.89. B, The infarct area of the brains was assessed by TTC staining. Five rats per group and these sections were from equivalent areas of the brains. SD (MCAo): 5.13; SD (MCAo + NC): 4.61; SD (MCAo + miR‐92b): 3.66. C, The brain water content was tested. Six rats per group. SD (Sham): 1.76; SD (MCAo): 1.62; SD (MCAo + NC): 1.61; SD (MCAo + miR‐92b): 1.25. D, The BBB permeability was assessed by the Evans Blue staining. Six rats per group. SD (Sham): 0.14; SD (MCAo): 1.38; SD (MCAo + NC): 1.17; SD (MCAo + miR‐92b): 0.66. E, The expressions of junctional proteins (occludin, ZO‐1, VE‐cadherin in brains) were assessed by Western blot. Six rats per group. F, The activity of SOD in the brain was assessed by ELISA kits. SD (Sham): 0.94; SD (MCAo): 0.66; SD (MCAo + NC): 0.78; SD (MCAo + miR‐92b): 0.48. G, The ROS production in the brain was assessed by the DCFH‐DA method. SD (Sham): 11.66; SD (MCAo): 19.39; SD (MCAo + NC): 13.57; SD (MCAo + miR‐92b): 8.83. *P  < 0.05, **P < 0.01 vs sham or MCAo + NC. Data are represented as the mean ± SD of three independent assays

To probe into the role of miR‐92b overexpression in ischaemic stroke, rats were injected with AAV vectors expressing miR‐92b to overexpress miR‐92b in brains and then underwent MCAo surgery to create an ischaemic stroke model. As displayed in Figure 2A, MACo rats overexpressed miR‐92b had a lower neurological score, prompting that miR‐92b overexpression ameliorated the neurological function of MCAo rats. The TTC staining results expounded that the cerebral infarction volume of MCAo rats was lessened by miR‐92b overexpression (Figure 2B). Besides, we tested infarction volume at 1, 2 and 3 hours after the model construction and assessed the correlation between miR‐92b and infarction volume. The results expounded that the infarction volume was raised with time, whereas miR‐92b expression was lessened, and miR‐92b was negatively correlated with the infarction volume (Figure S2A and B). Meanwhile, the brain water content and the BBB permeability of MCAo rats were both lessened by miR‐92b overexpression (Figure 2C and D), and the results expounded that Claudin‐5 protein level was lessened in the model group, whereas this lessening was reversed after the overexpression of miR‐92b (Figure S2C). Besides, we conducted the HE staining of brain slices to further observe the pathological changes of the brain and expounded that the brain injury in rats was ameliorated after the overexpression of miR‐92b (Figure S2E) and the brain slices without any staining were shown in Figure S3. The protein levels of endothelial junctional proteins (occluding, ZO‐1 and VE‐cadherin) in the brains of MCAo rats were raised by the overexpression of miR‐92b (Figure 2E). Furthermore, the activity of SOD was raised and the ROS production was lessened in the brains of MCAo rats by the forced expression of miR‐92b (Figure 2F and G). Collectively, these findings expounded that miR‐92b overexpression maintained the BBB integrity of ischaemic stroke rats.

### 3.3 Overexpression of miR‐92b raised the viability and lessened the permeability of OGD‐induced BMECs

**FIGURE 3:**
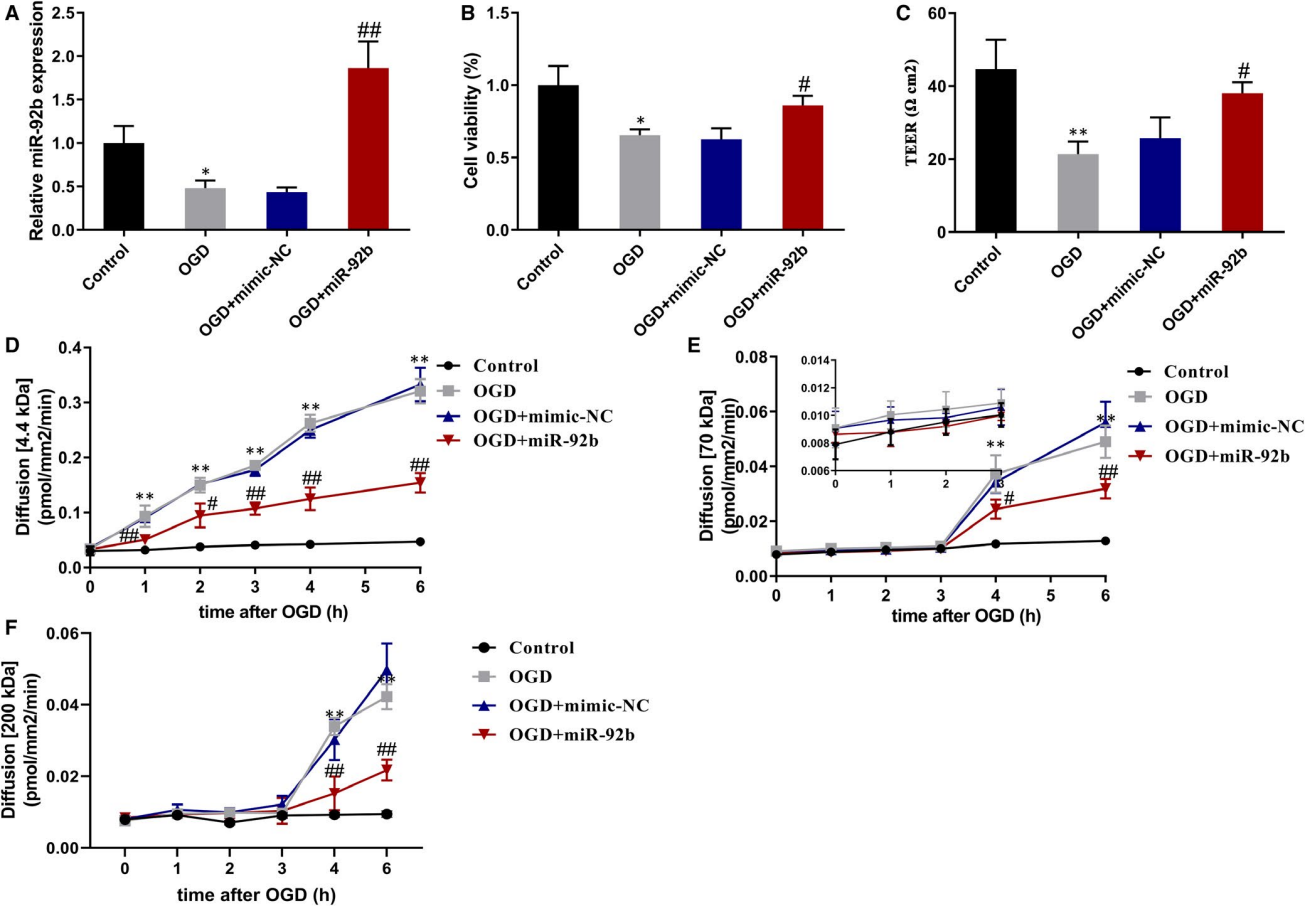
The effect of miR‐92b overexpression on the viability and permeability of OGD‐induced human BMECs. A‐C, hCMEC/D3 cells were transfected with miR‐92b mimic or negative control (mimic‐NC) and then induced by OGD for 6 h. A, The transfection efficiency of miR‐92b was assessed by qRT‐PCR. SD (Control): 0.20; SD (OGD): 0.09; SD (OGD + mimic‐NC): 0.06; SD (OGD + miR‐92b): 0.31. B, The viability of hCMEC/D3 cells was assessed by MTT assay. SD (Control): 0.13; SD (OGD): 0.04; SD (OGD + mimic‐NC): 0.08; SD (OGD + miR‐92b): 0.07. C, The permeability of hCMEC/D3 cells was assessed by the transendothelial electrical resistance (TEER). SD (Control): 8.02; SD (OGD): 3.51; SD (OGD + mimic‐NC): 5.69; SD (OGD + miR‐92b): 3.00. D‐E, hCMEC/D3 cells were transfected with miR‐92b mimic or negative control (NC) and then induced by OGD for 0, 1, 2, 3, 4, 5 and 6 h. The permeability of hCMEC/D3 cells was assessed by 4.4 kD TRITC‐dextran (D) and FITC‐dextran (E and F). D, SD (Control): 0.01; SD (OGD): 0.01; SD (OGD + mimic‐NC): 0.01; SD (OGD + miR‐92b): 0.01. E and F, SD (Control): 0.001; SD (OGD): 0.001; SD (OGD + mimic‐NC): 0.001; SD (OGD + miR‐92b): 0.001. *P < 0.05, **P < 0.01 vs control; #*P* < 0.05, ##*P* < 0.01 vs OGD + mimic‐NC. Data are represented as the mean ± SD of three independent assays

To probe into the influence of miR‐92b overexpression in the viability and permeability of OGD‐induced BMECs, BMECs were transfected with miR‐92b mimic to overexpress miR‐92b and followed by OGD‐induction, and then the viability and permeability of cells were tested. The transfection efficiency of miR‐92b was as displayed in Figure 3A. The results expounded that miR‐92b overexpression raised the viability of OGD‐induced BMECs (Figure 3B). Besides, we applied EZViableTM calcein‐AM cell viability assay kit (Fluorometric) to test cell viability, and the results expounded that OGD treatment lessened the number of viable cells, whereas this lessening was reversed after the overexpression of miR‐92b (Figure S1C). As displayed in Figure 3C, miR‐92b overexpression also raised the TEER of OGD‐induced BMECs, and the visual diagram of the TEER experiment was displayed in Figure S2D. Besides, the forced expression miR‐92b lessened the permeability of BMECs to 4.4 kD‐dextran at 1‐6 hours post‐OGD (Figure 3D) and 70 kD‐dextran at 4‐6 hours post‐OGD (Figure 3E) and 200 kD‐dextran at 4‐6 hours post‐OGD (Figure 3F). Thus, these results expounded that the overexpression of miR‐92b raised the viability and lessened the permeability of OGD‐induced BMECs.

### 3.4 miR‐92b negatively regulated NOX4 expression in BMECs

**FIGURE 4:**
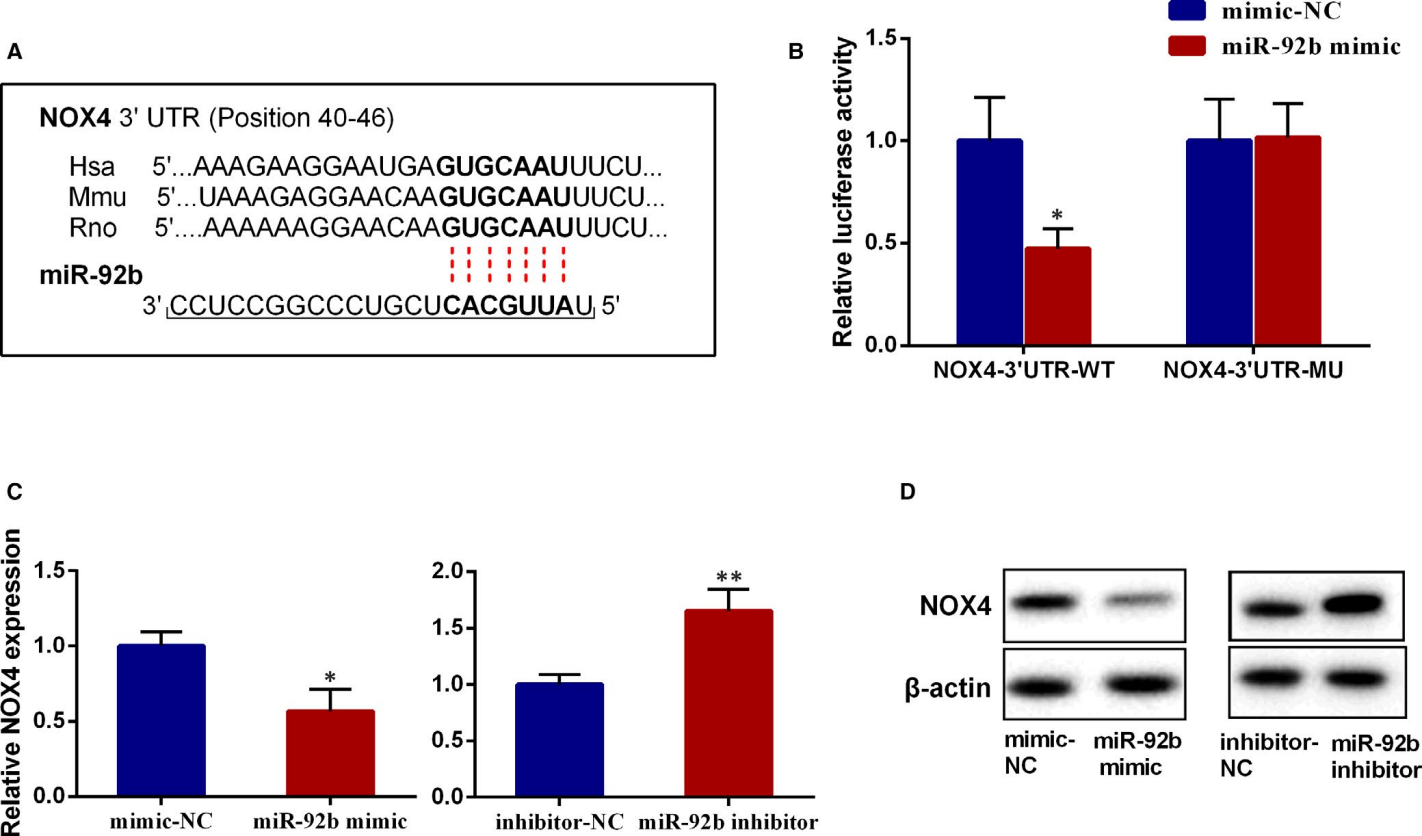
The effect on miR‐92b on the expression of NOX4. A, The binding sites of miR‐92b on the 3′‐UTR of NOX4. B, The wild‐type (WT) sequence of NOX4‐3′‐UTR (NOX4‐3′‐UTR‐WT) and mutated (MU) sequence of NOX4‐3′‐UTR (NOX4‐3′‐UTR‐MU) were subcloned into the upstream of the luciferase reporter gene in a pGL3 plasmid. Each recombinant vector was co‐transfected with miR‐92b mimic (or negative control: mimic‐NC) into 293T cells. Then, the luciferase activity was assessed by the Dual‐Luciferase Reporter Assay System. SD (NOX4‐3′‐UTR‐WT‐mimic‐NC): 0.21; SD (NOX4‐3′‐UTR‐WT‐miR‐92b mimic): 0.10; SD (NOX4‐3′‐UTR‐MU‐mimic‐NC): 0.20; SD (NOX4‐3′‐UTR‐MU‐miR‐92b mimic): 0.17. C and D, hCMEC/D3 cells were transfected with miR‐92b mimic, miR‐92b inhibitor or their negative controls (mimic‐NC and inhibitor NC), respectively. The mRNA level (C) and protein level (D) of NOX4 were tested by qRT‐PCR and Western blot, respectively. C, SD (mimic‐NC): 0.10; SD (miR‐92b mimic): 0.15; SD (inhibitor‐NC): 0.09; SD (miR‐92b inhibitor): 0.20. *P < 0.05, **P < 0.01 vs mimic‐NC or inhibitor‐NC. Data are represented as the mean ± SD of three independent assays

As displayed in Figure 4A, the 3′‐UTR sequences of NOX4 in humans, rats and mice are highly conserved and all had binding sites of miR‐92b. To probe into whether miR‐92b targeted NOX4, we conducted the dual‐luciferase reporter assay in 293T cells. The results expounded that miR‐92b overexpression lessened the luciferase activity of WT NOX4‐3′‐UTR, whereas had no significant influence on the luciferase activity of MU NOX4‐3′‐UTR (Figure 4B). To further probe into the regulation of miR‐92b on NOX4 expression in hCMEC/D3 cells, we tested the expression of NOX4 in miR‐92b overexpressed or silenced hCMEC/D3 cells. We discovered that miR‐92b overexpression lessened the mRNA and protein levels of NOX4, and miR‐92b silence raised the mRNA and protein levels of NOX4 (Figure 4C and D). Therefore, those results expounded that miR‐92b targeted NOX4 and negatively regulated their expression in hCMEC/D3 cells.

### 3.5 NOX4 mediated the effect of miR‐92b on the viability and permeability of OGD‐induced BMECs

**FIGURE 5:**
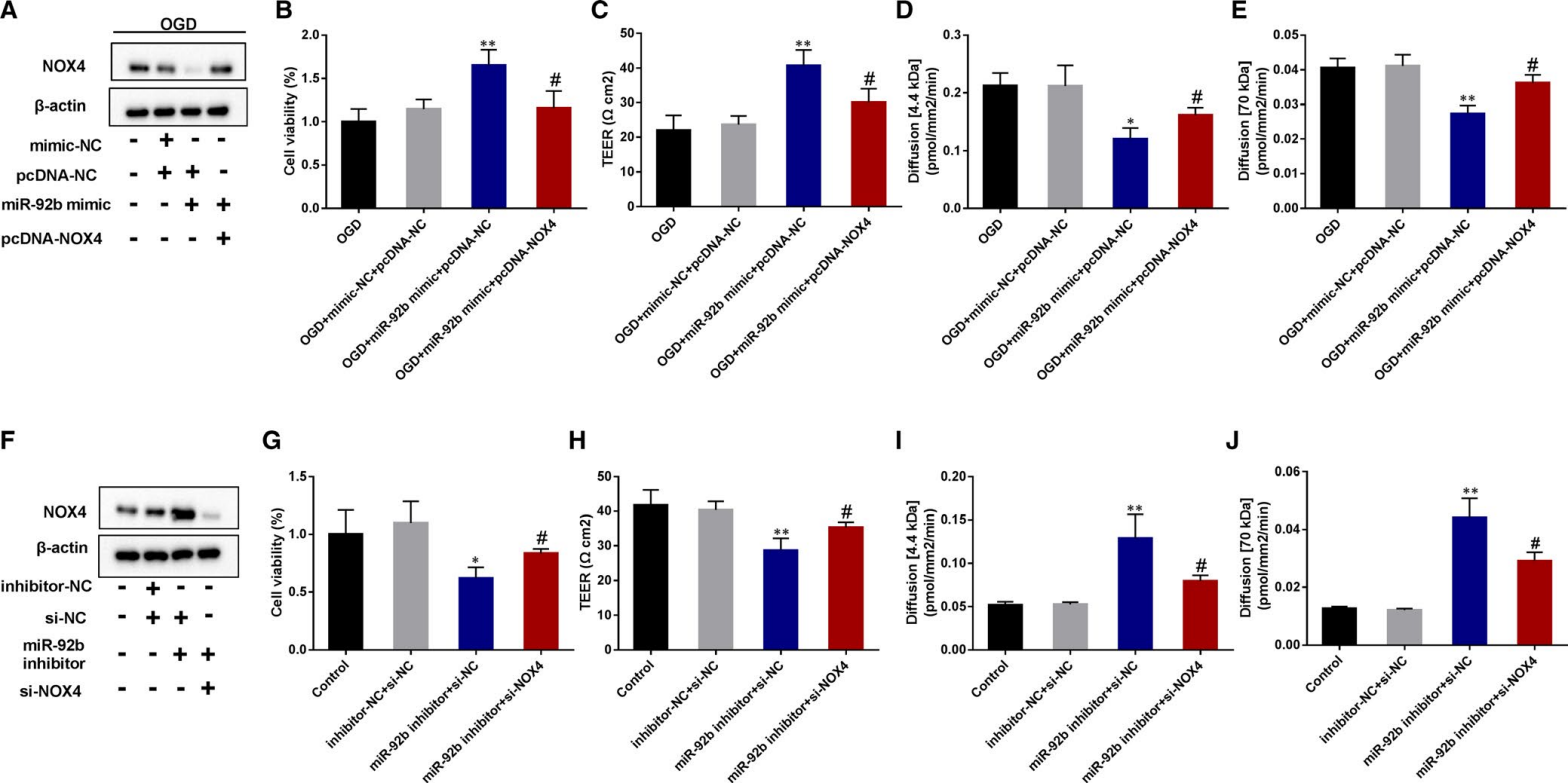
NOX4 mediated the effect of miR‐92b overexpression on the viability and permeability of OGD‐induced human BMECs. A‐E, hCMEC/D3 cells were co‐transfected with mimic‐NC and pcDNA, miR‐92b mimic and pcDNA, or miR‐92b mimic and pcDNA‐NOX4 and then induced by OGD for 4 h. A, The protein level of NOX4 was tested by Western blot. B, The viability of hCMEC/D3 cells was assessed by MTT assay. SD (OGD): 0.21; SD (OGD + mimic‐NC + pcDNA‐NC): 0.19; SD (OGD + miR‐92b mimic + pcDNA‐NC): 0.10; SD (OGD + miR‐92b mimic + pcDNA‐NOX4): 0.04. C, The transendothelial electrical resistance of hCMEC/D3 cells. SD (OGD): 4.51; SD (OGD + mimic‐NC + pcDNA‐NC): 2.52; SD (OGD + miR‐92b mimic + pcDNA‐NC): 3.51; SD (OGD + miR‐92b mimic + pcDNA‐NOX4): 1.53. D and E, The permeability of hCMEC/D3 cells was assessed by 4.4 kD TRITC‐dextran (D) and FITC‐dextran (E). D, SD (OGD): 0.004; SD (OGD + mimic‐NC + pcDNA‐NC): 0.003; SD (OGD + miR‐92b mimic + pcDNA‐NC): 0.03; SD (OGD + miR‐92b mimic + pcDNA‐NOX4): 0.01. E, SD (OGD): 0.001; SD (OGD + mimic‐NC + pcDNA‐NC): 0.001; SD (OGD + miR‐92b mimic + pcDNA‐NC): 0.01; SD (OGD + miR‐92b mimic + pcDNA‐NOX4): 0.003. F‐J, hCMEC/D3 cells were co‐transfected with inhibitor‐NC and si‐NC, miR‐92b inhibitor and si‐NC or miR‐92b inhibitor and si‐NOX4 and then induced by OGD for 4 h. F, The protein level of NOX4 was tested by Western blot. G, The viability of hCMEC/D3 cells was assessed by MTT assay. SD (OGD): 0.15; SD (OGD + inhibitor‐NC + pcDNA‐NC): 0.11; SD (OGD + miR‐92b inhibitor + si‐NC): 0.18; SD (OGD + miR‐92b inhibitor + si‐NOX4): 0.19. H, The transendothelial electrical resistance of hCMEC/D3 cells. SD (OGD): 4.36; SD (OGD + inhibitor‐NC + pcDNA‐NC): 2.52; SD (OGD + miR‐92b inhibitor + si‐NC): 4.51; SD (OGD + miR‐92b inhibitor + si‐NOX4): 4.00. I and J, The permeability of hCMEC/D3 cells was assessed by 4.4 kD TRITC‐dextran (I) and FITC‐dextran (J). I, SD (OGD): 0.02; SD (OGD + inhibitor‐NC + pcDNA‐NC): 0.04; SD (OGD + miR‐92b inhibitor + si‐NC): 0.02; SD (OGD + miR‐92b inhibitor + si‐NOX4): 0.01. J, SD (OGD): 0.003; SD (OGD + inhibitor‐NC + pcDNA‐NC): 0.003; SD (OGD + miR‐92b inhibitor + si‐NC): 0.002; SD (OGD + miR‐92b inhibitor + si‐NOX4): 0.002. *P < 0.05, **P < 0.01 vs OGD + mimic + pcDNA or inhibitor‐NC + si‐NC; #*P* < 0.05, ##*P* < 0.01 vs OGD + miR‐92b mimic + pcDNA or miR‐92b inhibitor + si‐NC. Data are represented as the mean ± SD of three independent assays

We then probed into whether NOX4 mediated the influence of miR‐92b in the viability and permeability of OGD‐induced BMECs. The results expounded that miR‐92b overexpression lessened the protein level of NOX4 in OGD‐induced BMECs, whereas pcDNA‐NOX4 abolished this effect (Figure 5A). The viability and TEER of OGD‐induced BMECs were raised by miR‐9b overexpression, whereas NOX4 overexpression reversed that trend (Figure 5B and C). The permeability of OGD‐induced BMECs to 4.4 kD‐dextran and 70 kD‐dextran was lessened by miR‐92b overexpression, whereas those trends were abrogated by NOX4 overexpression (Figure 5D and E). Meanwhile, the results expounded that miR‐92b knockdown raised the protein level of NOX4 in OGD‐induced BMECs, whereas si‐NOX4 abolished this effect (Figure 5F). The viability and TEER of OGD‐induced BMECs were lessened by miR‐9b silence, whereas NOX4 knockdown reversed that trend (Figure 5G and H). The permeability of OGD‐induced BMECs to 4.4 kD‐dextran and 70 kD‐dextran was raised by miR‐92b silence, whereas those trends were abrogated by NOX4 knockdown (Figure 5I and J). Taken together, these findings expounded that NOX4 mediated the influence of miR‐92b in the viability and permeability of OGD‐induced BMECs.

### 3.6 Transcription factor Foxo1 restrained miR‐92b expression in BMECs

**FIGURE 6:**
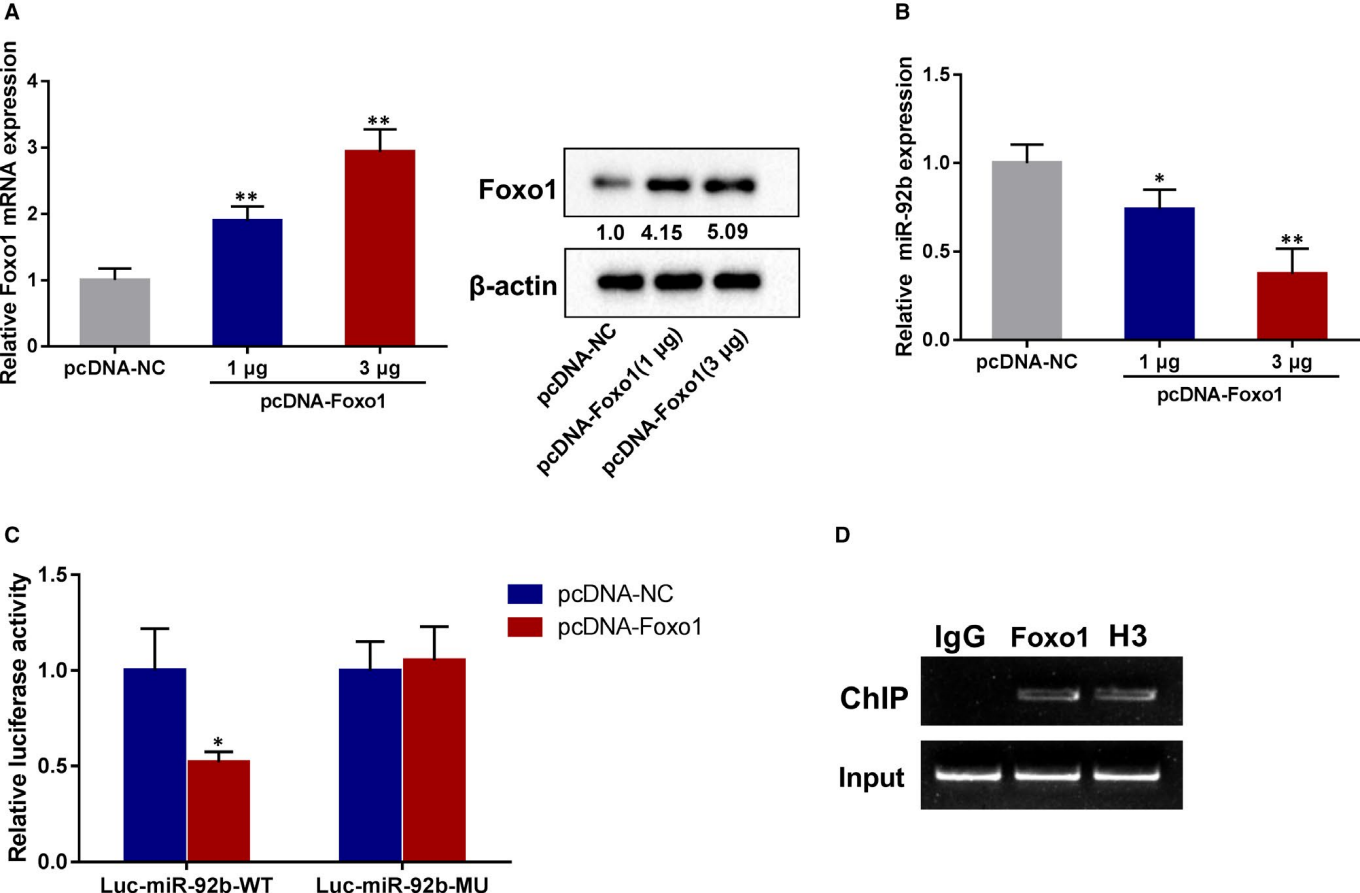
The effect of Foxo1 on miR‐92b expression in human BMECs. hCMEC/D3 cells were transfected with 1 µg or 3 µg pcDNA‐Foxo1. A, The transfection efficiency of pcDNA‐Foxo1 was assessed by qRT‐PCR and Western blot. SD (pcDNA‐NC): 0.18; SD (pcDNA‐Foxo1‐1 µg): 0.22; SD (pcDNA‐Foxo1‐3 µg): 0.34. B, The expression of miR‐92b was assessed by qRT‐PCR. SD (pcDNA‐NC): 0.11; SD (pcDNA‐Foxo1‐1 µg): 0.11; SD (pcDNA‐Foxo1‐3 µg): 0.14. C, The WT promoter sequence of miR‐92b (miR‐92b‐WT) and MU promoter sequence of miR‐92b (miR‐92b‐MU) were subcloned into the upstream of a luciferase reporter gene in a pGL3 plasmid. Each recombinant vector was co‐transfected with pcDNA‐Foxo1 (or negative control: pcDNA) into 293T cells. Then, the luciferase activity was assessed by the Dual‐Luciferase Reporter Assay System. SD (Luc‐miR‐92b‐WT‐pcDNA‐NC): 0.22; SD (Luc‐miR‐92b‐WT‐pcDNA‐Foxo1): 0.06. D, The enrichment of Foxo1 in the promoter of miR‐92b was assessed by CHIP assay. *P < 0.05, **P < 0.01 vs pcDNA‐NC. Data are represented as the mean ± SD of three independent assays

Previous studies have expounded that transcription factors influence the transcriptional expression of miRNAs under physiological and pathological conditions.^29^, ^30^ In view of the bioinformatics analysis, we noticed that there were potential binding sites of Foxo1 in the promoter of miR‐92b. More momentously, Foxo1 is activated by phosphorylation in the ischaemic brains and has been confirmed to be bound up with the BBB dysfunction after ischaemic stroke.^31^ Given this, we speculated that Foxo1 influenced the transcriptional expression of miR‐92b in ischaemic stroke. To probe into the role of Foxo1 in the regulation of miR‐92b, we tested the expression of miR‐92b in Foxo1 overexpressed hCMEC/D3 cells. As displayed in Figure 6A, the transfection efficiency of pcDNA‐Foxo1 was tested by qRT‐PCR and Western blot. miR‐92b expression was lessened by Foxo1 overexpression (Figure 6B). To confirm the interaction between miR‐92b and Foxo1, we conducted dual‐luciferase reporter and ChIP assays in hCMEC/D3 cells. The results expounded that the overexpression of Foxo1 lessened the luciferase activity of the WT miR‐92b promoter, whereas it had no significant effect on the MU miR‐92b promoter (Figure 6C). Additionally, the results expounded that the miR‐92b promoter was more enriched in the Foxo1‐immunoprecipitated products than that in IgG‐immunoprecipitated products (Figure 6D). Therefore, the present data expounded that Foxo1 restrained miR‐92b expression in BMECs.

### 3.7 OGD lessened the viability and raised the permeability of BMECs through the Foxo1/miR‐92b pathway

**FIGURE 7:**
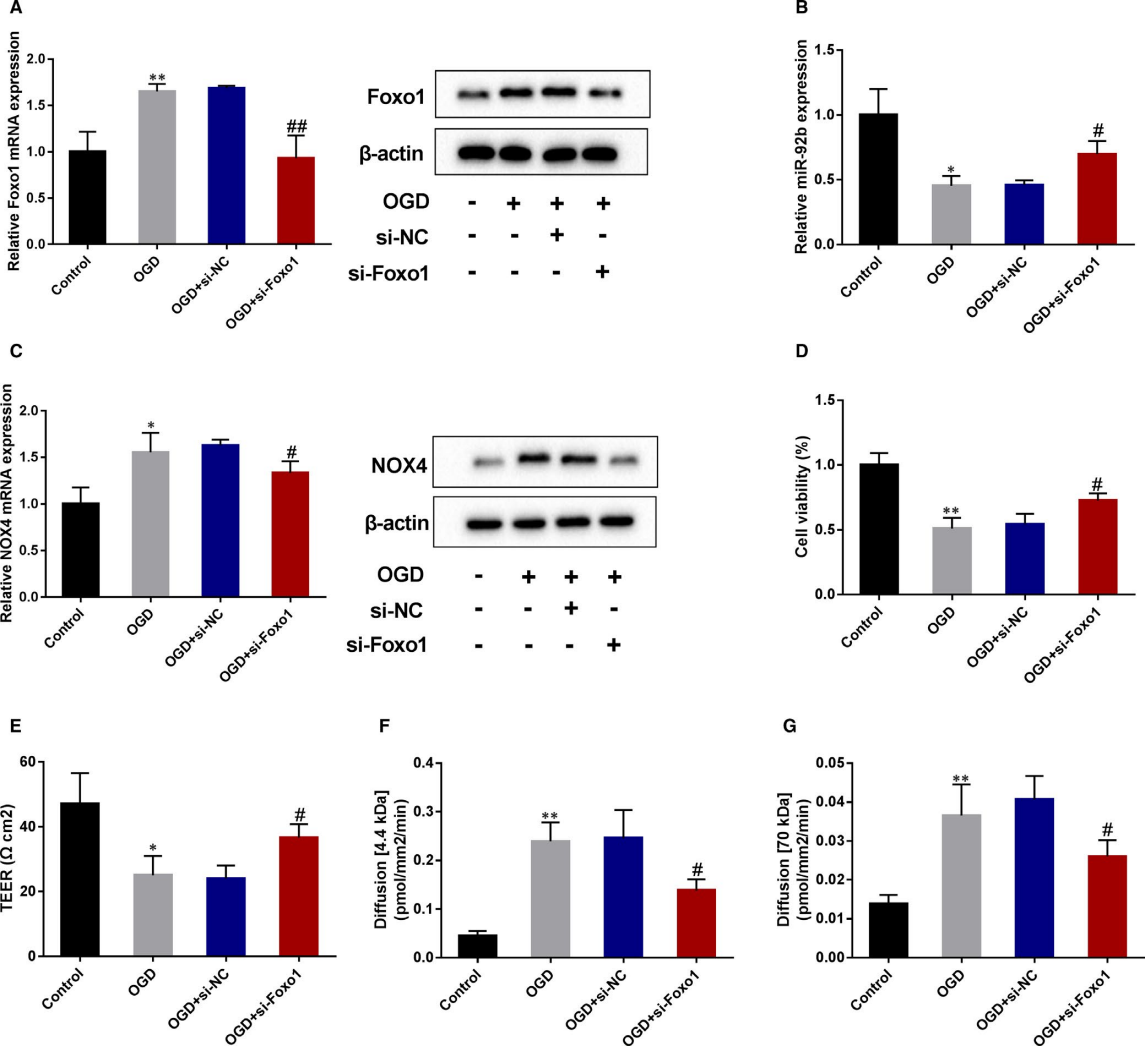
The effect of Foxo1 knockdown on the expressions of miR‐92b and NOX4, viability and permeability of OGD‐induced human BMECs. hCMEC/D3 cells were transfected with si‐Foxo1 or negative control (si‐NC) and then induced by OGD. A, The transfection efficiency of si‐Foxo1 was assessed by qRT‐PCR and Western blot. SD (Control): 0.22; SD (OGD): 0.08; SD (OGD + si‐NC): 0.03; SD (OGD + si‐Foxo1): 0.25. B, The expression of miR‐92b was assessed by qRT‐PCR. SD (Control): 0.20; SD (OGD): 0.08; SD (OGD + si‐NC): 0.04; SD (OGD + si‐Foxo1): 0.10. C, The expression of NOX4 was assessed by qRT‐PCR and Western blot. SD (Control): 0.18; SD (OGD): 0.21; SD (OGD + si‐NC): 0.06; SD (OGD + si‐Foxo1): 0.13. D, Cell viability was assessed by MTT assay. SD (Control): 0.09; SD (OGD): 0.08; SD (OGD + si‐NC): 0.08; SD (OGD + si‐Foxo1): 0.06. E, The TERR fo cells. SD (Control): 9.54; SD (OGD): 6.00; SD (OGD + si‐NC): 4.00; SD (OGD + si‐Foxo1): 4.16. F and G, The permeability of hCMEC/D3 cells was assessed by 4.4 kD TRITC‐dextran (F) and FITC‐dextran (G). F, SD (Control): 0.01; SD (OGD): 0.04; SD (OGD + si‐NC): 0.06; SD (OGD + si‐Foxo1): 0.02. G, SD (Control): 0.002; SD (OGD): 0.01; SD (OGD + si‐NC): 0.01; SD (OGD + si‐Foxo1): 0.004. *P < 0.05, **P < 0.01 vs control; #*P* < 0.05, ##*P* < 0.01 vs OGD + si‐NC. Data are represented as the mean ± SD of three independent assays

Finally, we probed into whether OGD lessened the viability and raised the permeability of BMECs through the Foxo1/miR‐92b axis. As displayed in Figure 7A, the OGD‐induction increased Foxo1 expression in BMECs and si‐Foxo1 reversed the effect. The OGD‐induction lessened miR‐92b expression and raised NOX4 expression in BMECs, and the silence of Foxo1 reversed those effects (Figure 7B and C). The viability and TEER of BMECs were lessened by OGD‐induction, whereas the knockdown of Foxo1 abrogated those trends (Figure 7D and E). Moreover, OGD‐induction raised the permeability of BMECs to 4.4 kD‐dextran, and 70 kD‐dextran and Foxo1 silence reversed that impacts (Figure 7F and G). Hence, those findings expounded that OGD lessened the viability and raised the permeability of BMECs through the Foxo1/miR‐92b axis.

## 4 DISCUSSIONS

Increasingly, studies expounded that miRNAs are bound up with the BBB damage after ischaemic strokes, such as miR‐126‐3p, miR‐98 and miR‐132.^32^, ^33^, ^34^ miR‐92b is a highly conserved miRNA in humans, mice and rats. The previous pieces of literature have expounded that miR‐92b is momentous in boosting the muscle development of *Drosophila* and in restraining the production of intermediate cortical progenitors in mouse embryonic brain.^35^, ^36^ It has been proven that miR‐92b is abnormally expressed in many diseases, such as alcoholic hepatitis, smoking‐related interstitial fibrosis, heart failure and cancers.^37^, ^38^, ^39^, ^40^ At present, the biological role of miR‐92b in cancers has been well established, including hepatocellular carcinoma, breast cancer, oesophageal cancer^40^ and *et al*. However, the roles of miR‐92b in other diseases remain largely unknown. In the current study, we discovered that miR‐92b expression was lessened in the ischaemic brains of rats and in the OGD‐induced BMECs, implying that miR‐92b might be bound up with ischaemic stroke. This finding was similar to the previously reported literature,^41^ and our study also indicated that miR‐92b could express in all of the five types of cells and predominantly express in BMECs, pericytes and the astrocytes, and this is a small innovation in our research. Our further study expounded that the overexpression of miR‐92b ameliorated the neurological function, lessened infarct volume of brains and relieved BBB damage of rat brains after ischaemic stroke. Meanwhile, the *in vitro* model also expounded that miR‐92b overexpression raised the viability and permeability of OGD‐induced BMECs. Hence, those findings expounded that miR‐92b protected the integrity of BBB after ischaemic stroke.

As known, miRNAs usually exert their biological functions by regulating the expression of target genes.^16^ NOX4 is an NADPH oxidase and has been confirmed to dedicate to the BBB damage after ischaemic stroke.^11^, ^12^ Many evidence expounded that miRNAs can target NOX4 in many diseases. For instance, miR‐146a can alleviate high glucose/thrombin‐induced endothelial inflammation in diabetic atherothrombosis via targeting NOX4 expression^42^; miR‐99a‐5p can restrain the proliferation, migration and invasion of oral carcinoma cells by targeting NOX4 expression^17^; miR‐423‐5p can attenuate high glucose‐induced podocyte injury in diabetic nephropathy by targeting NOX4 expression.^18^ Given the bioinformatics analysis, we discovered binding sites of miR‐92b on the 3′‐UTR of NOX4, implying that NOX4 might also be a target of miR‐92b. To verify this, we conducted a luciferase reporter gene and the results expounded that miR‐92b could target NOX4. The expression of NOX4 in BMECs could be negatively regulated by miR‐92b. This was similar to the findings of previous study,^43^ but our novelty mainly included the following two points: (1) In our study, we directly proved the targeted binding of miR‐92b and NOX4, whereas the literature provided that whether miR‐92b‐3p‐regulated NOX4 was only verified by the lost function of miR‐92b‐3p. We conducted direct studies through dual‐luciferase reporter assay, and the study of the binding of the two was more direct; (2) The regulation of NOX4 by miR‐92b in disease‐related cerebral vascular endothelial cells was confirmed by gain or lost function, and this was the first study of these two molecules in these cells. More momentously, our further results expounded that NOX4 mediated the effects of miR‐92b on the viability and permeability of OGD‐induced BMECs. Therefore, our present data expounded that miR‐92b restrained the BBB damage after ischaemic stroke by lessening NOX4 expression.

Foxo1 is a member of ‘O’ class forkhead transcription factors and has been reported to be implicated in various diseases, such as diabetes, myocardial glucose oxidation and cancers.^44^, ^45^, ^46^ Recent studies expounded that Foxo1 is also bound up with ischaemic stroke. Ma *et al* discovered that Foxo1 mediates the effect of selenium nanoparticles on the inflammation of the rat model of ischaemic stroke.^47^ Kou *et al* expounded that Foxo1 mediates the effect of estradiol on neuronal cell apoptosis in the rat model of ischaemic stroke.^48^ Lv *et al* expounded that Foxo1 mediates the effect of salvianolic acid B on inflammation and apoptosis.^49^ Besides, Foxo1 has been proven to be bound up with BBB damage after ischaemic stroke.^50^ In this study, we expounded that Foxo1 expression was raised *in vitro* model of damaged BBB induced by OGD. The knockdown of Foxo1 raised the viability and lessened the permeability of OGD‐induced BMECs. Therefore, our results expounded that Foxo1 boosted OGD‐induced BBB damage.

It has been proven that miRNAs can be influenced by various transcription factors under physiological and pathological conditions. For instance, miR‐191 is induced by transcription factor HIF‐1α in hepatic ischaemia/reperfusion injury^29^; miR‐545‐3p is stimulated by transcription factor SP1 in osteoporosis.^30^ A previous study also expounded that miR‐424 is restrained by transcription factor Foxo1 in the osteogenic differentiation of mesenchymal stem cells.^51^ Given the bioinformatics analysis, we noticed that there were potential binding sites of Foxo1 in the promoter of miR‐92b, implying that miR‐92b might be regulated by Foxo1. The overexpression of Foxo1 restrained the expression of miR‐92b in BMECs. We then conducted the luciferase reporter gene and CHIP assays to verify the interaction between Foxo1 and miR‐92b. The results expounded that Foxo1 could bind to the promoter of miR‐92b. Furthermore, the knockdown of Foxo1 raised the expression of miR‐92b in OGD‐induced BMECs. Thus, those results expounded that the lessening of miR‐92b in OGD‐induced BMECs was induced by Foxo1.

In conclusion, the present study expounded that miR‐92b expression was lessened by Foxo1 after ischaemic stroke and that the lessening of miR‐92b facilitated the BBB damage by targeting NOX4. Besides, the limitation of this study was that there was no clinical application of Foxo1 in ischaemic stroke, and we would continue to probe into the clinical application value of Foxo1 and its specific inhibitors to further ameliorate this study.

## CONFLICT OF INTEREST

All authors declare that they have no conflicts of interest in this work.

## AUTHOR CONTRIBUTIONS

**Jian Shen:** Conceptualization (equal); Formal analysis (equal); Writing‐original draft (equal). **Ganglei Li:** Data curation (equal). **Yu Zhu:** Formal analysis (equal). **Qingsheng Xu:** Formal analysis (equal). **Hengjun Zhou:** Formal analysis (equal). **Kangli Xu:** Formal analysis (equal). **Kaiyuan Huang:** Formal analysis (equal). **Jianwei Pan:** Investigation (equal); Writing‐review & editing (equal). **Renya Zhan:** Investigation (equal).

## Supporting information

Fig S1

Fig S2

Fig S3
