# Supplementary figures and images for: Foxo1‐induced miR‐92b down‐regulation promotes blood‐brain barrier damage after ischaemic stroke by targeting NOX4
Source: J Cell Mol Med. 2021 May 6;25(11):5269–82. doi: 10.1111/jcmm.16537 (PMC8178288; doi:10.1111/jcmm.16537)

**A**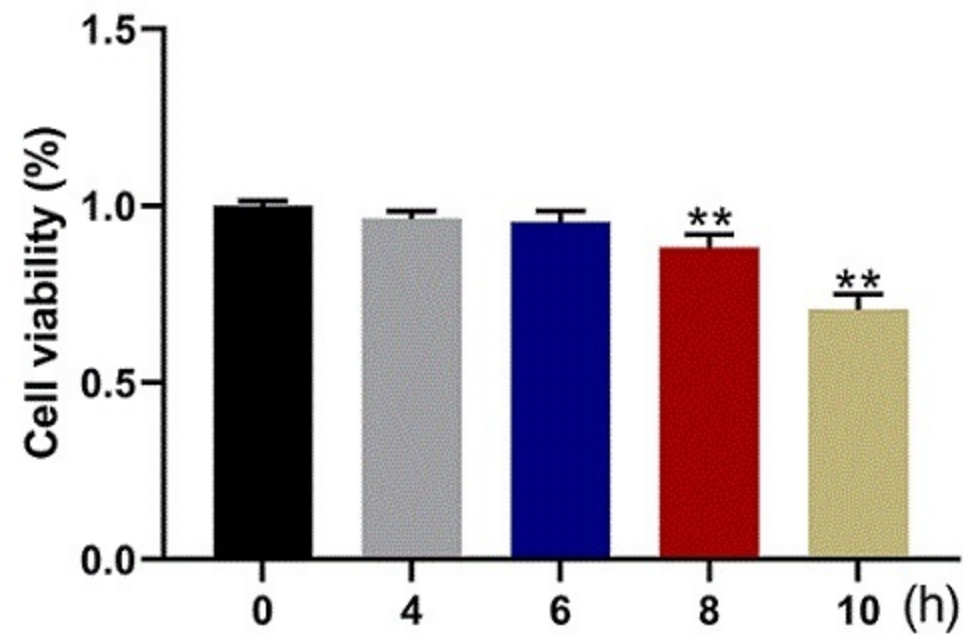**B**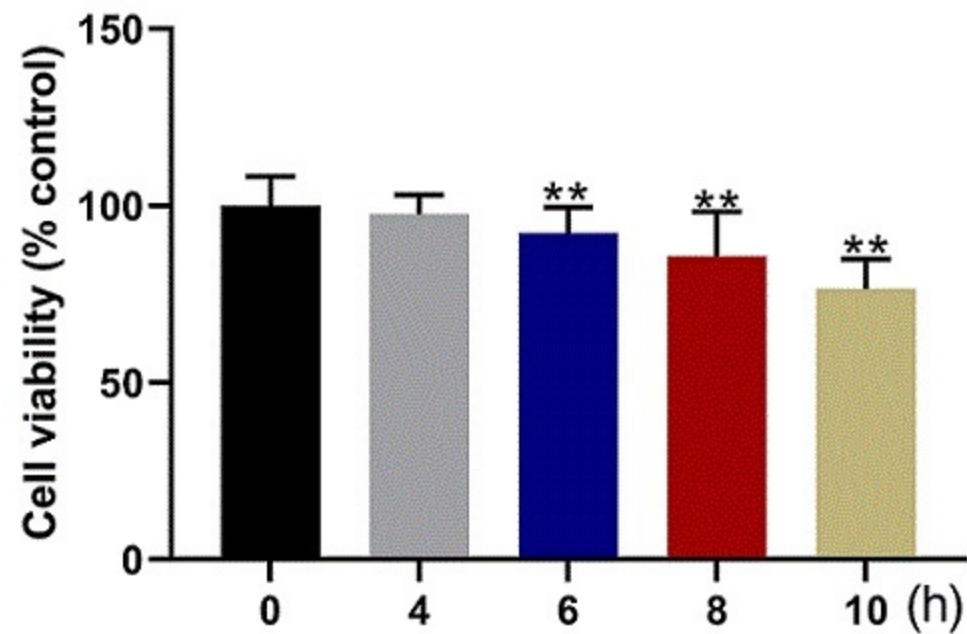**C**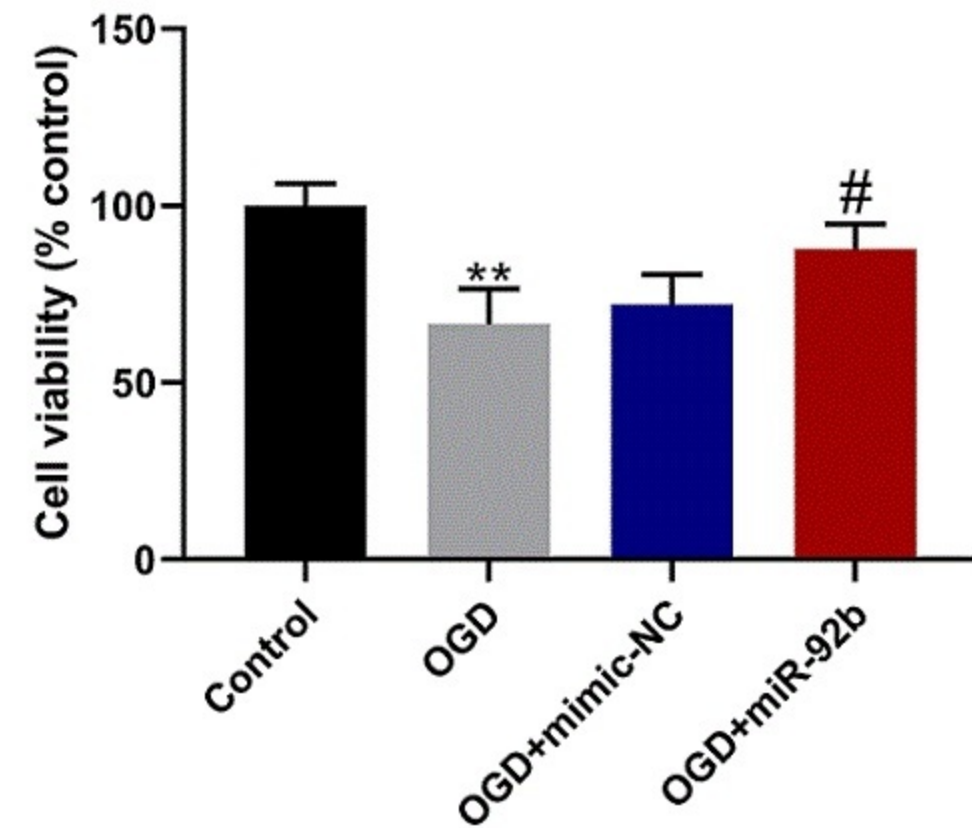

Supplement: Supplementary file 1 — Fig S1 [file JCMM-25-5269-s003.pdf]

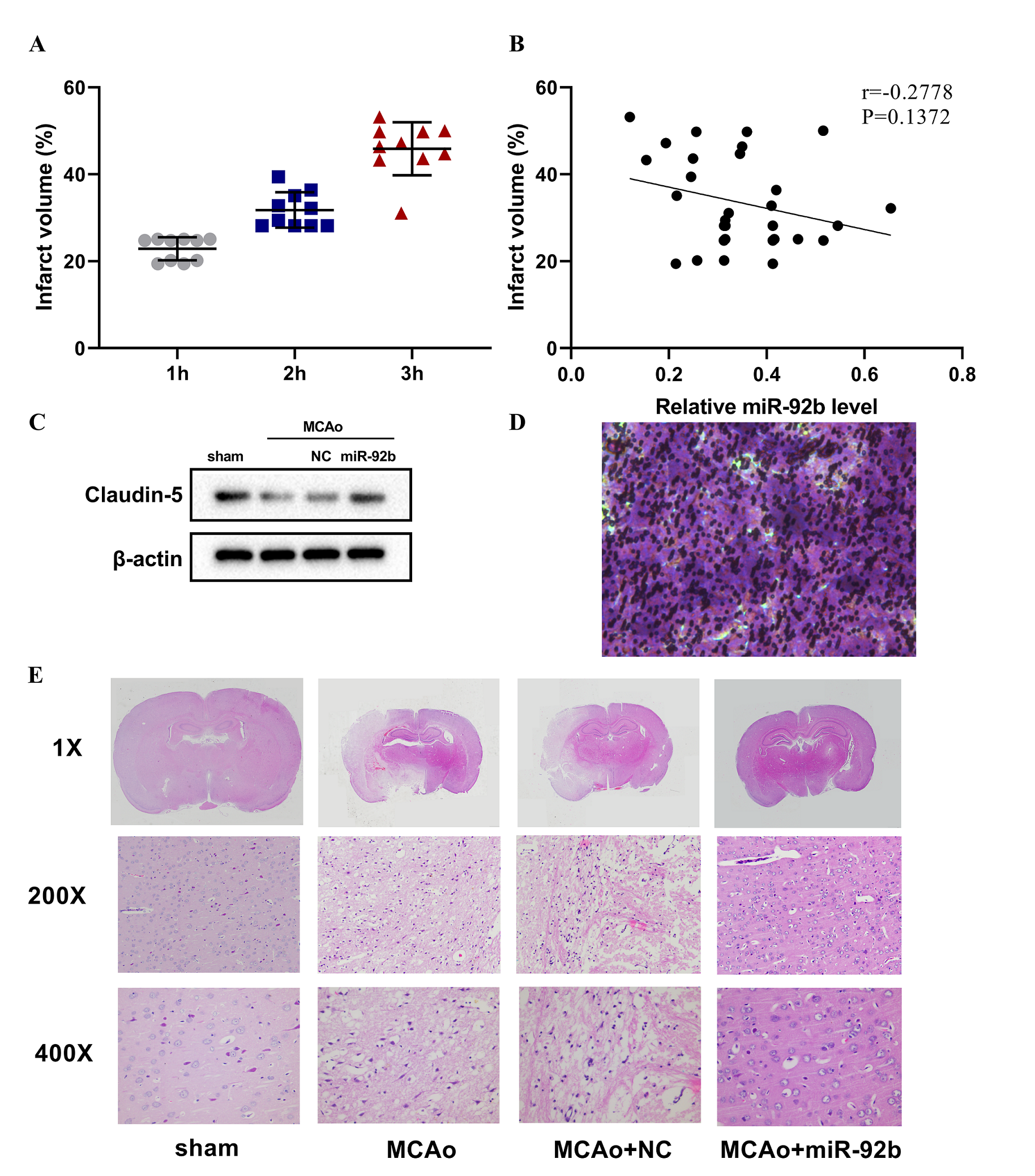

Supplement: Supplementary file 2 — Fig S2 [file JCMM-25-5269-s002.tif]

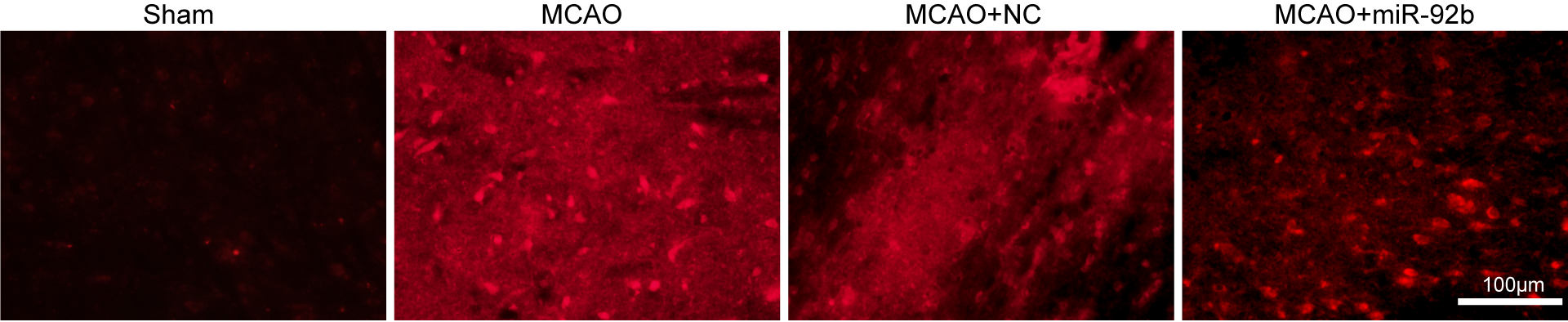

Supplement: Supplementary file 3 — Fig S3 [file JCMM-25-5269-s001.tif]
